# Activin a suppresses peripheral CD8^+^ T lymphocyte activity in acute-phase Kawasaki disease

**DOI:** 10.1186/s12865-021-00407-x

**Published:** 2021-02-23

**Authors:** Qian Wu, Ruohang Weng, Yongbin Xu, Linlin Wang, Yanyan Huang, Jun Yang

**Affiliations:** 1 State Key Laboratory of Chemical Oncogenomics, Key Laboratory of Chemical Genome, Peking University, Shenzhen Graduate School, School of Chemical Biology & Biotechnology, Shenzhen, 518055 China; 2 Department of Rheumatology and Immunology, Shenzhen Children’s Hospital, 7019 Yitian Road, Shenzhen, 518026 China

**Keywords:** Kawasaki disease, Activin type IIA receptor, Peripheral lymphocytes, Activin a

## Abstract

**Background:**

Kawasaki disease is an autoimmune disease characterized by systemic vasculitis of unknown aetiology and most commonly occurs in children under 5 years old. Previous studies have found that the over-activation of lymphocytes is an important mechanism of Kawasaki disease. Activin A, also known as immunosuppressive factor P, is a multifunctional growth and transforming factor. However, whether activin A is involved in the regulation of peripheral lymphocytes activity in Kawasaki disease is unclear. Thus, we aimed to investigate the effect of activin A on the activity of peripheral lymphocytes in acute-phase Kawasaki disease.

**Methods:**

Seven patients with Kawasaki disease and seven healthy controls were studied. Peripheral blood lymphocytes were isolated by Ficoll density gradient centrifugation. The activation of CD4^+^ and CD8^+^ T cells and CD19^+^ B cells was investigated by flow cytometry. The expression of activin type IIA receptors was investigated by flow cytometry.

**Results:**

Immune imbalance in CD4 and CD8 lymphocytes were detected in acute-phase Kawasaki disease. The expression of activin type IIA receptors on CD8^+^ T cells and CD19^+^ B cells was increased in acute-phase Kawasaki disease and decreased following treatment with activin A. Activin A suppressed the expression of CD25 and CD69 on CD8^+^ T cells and the expression of CD69 on CD19^+^ B cells.

**Conclusions:**

The expression of activin type IIA receptor was increased on CD8^+^ T cells and CD19^+^ B cells in Kawasaki disease. Activin A suppressed the expression of CD25, CD69 and activin type IIA receptors on peripheral CD8^+^ T lymphocyte. Activin A plays different roles in different lymphocyte subsets and suppresses peripheral CD8^+^ T lymphocyte activity in acute-phase Kawasaki disease.

**Supplementary Information:**

The online version contains supplementary material available at 10.1186/s12865-021-00407-x.

## Background

Kawasaki disease (KD) is an acute, autoimmune-like, self-limited vasculitis disease that seriously threatens cardiac function. However, its aetiology and pathogenesis remain unclear. Previous studies suggest that over-activation of the immune system plays important roles in the occurrence and development of Kawasaki disease [1, 2]. The response of the disease to intravenous immunoglobulin therapy is associated with the degree of activation of CD8^+^ T cells [3]. T cells may be activated in Kawasaki disease patients resistant to both initial and additional IVIG. Increased expression of HLA-DR in CD4+ T cells and CD8+ T cells was associated with IVIG resistance [4, 5]. Histopathologic studies of KD vasculitis lesions have demonstrated characteristic T cell infiltration and an abundance of CD8+ T cells [6]. Th17- and Treg-related cytokine and mRNA expression are associated with acute and resolving Kawasaki disease [4]. A paucity of IgA^+^ peripheral B cells in acute-phase Kawasaki disease has been reported, which continues through convalescence. However, some studies have found no changes in B cell subgroups in the acute and convalescent phases but have found increases in CD69^+^ natural killer and γδ T cells [5, 6]. Peripheral lymphocytes are imbalanced and abnormally activated in the acute phase of Kawasaki disease.

Activin A, also known as immunosuppressive factor P, belongs to the TGF-β superfamily. Extensive research over the past decades has illuminated the fundamental roles of activin A in a variety of biological processes, including embryonic development, cell proliferation, tissue fibrosis and the secretion of inflammatory mediators [7–10]. Activated immune cells such as T cells, B cells, monocytes, dendritic cells and mast cells synthesize and secrete activin A. However, the effect of activin A on peripheral lymphocytes in acute-phase Kawasaki disease has not been reported. Therefore, this study aimed to investigate the effect of activin A on the activity of peripheral lymphocytes in acute-phase Kawasaki disease.

In this study, we found that the expression of activin type IIA receptor (ActRIIA) on over-activated peripheral lymphocytes was increased. Furthermore, we found that ActRIIA, CD25 and CD69 expression was decreased on CD8^+^ T lymphocyte stimulated with activin A in vitro. This study suggests that peripheral lymphocytes were activated in acute-phase Kawasaki disease. In addition, activin A downregulated the activity of CD8^+^ T lymphocyte and the expression of ActRIIA.

## Results

### Immune imbalance in acute-phase Kawasaki disease

**Fig. 1:**
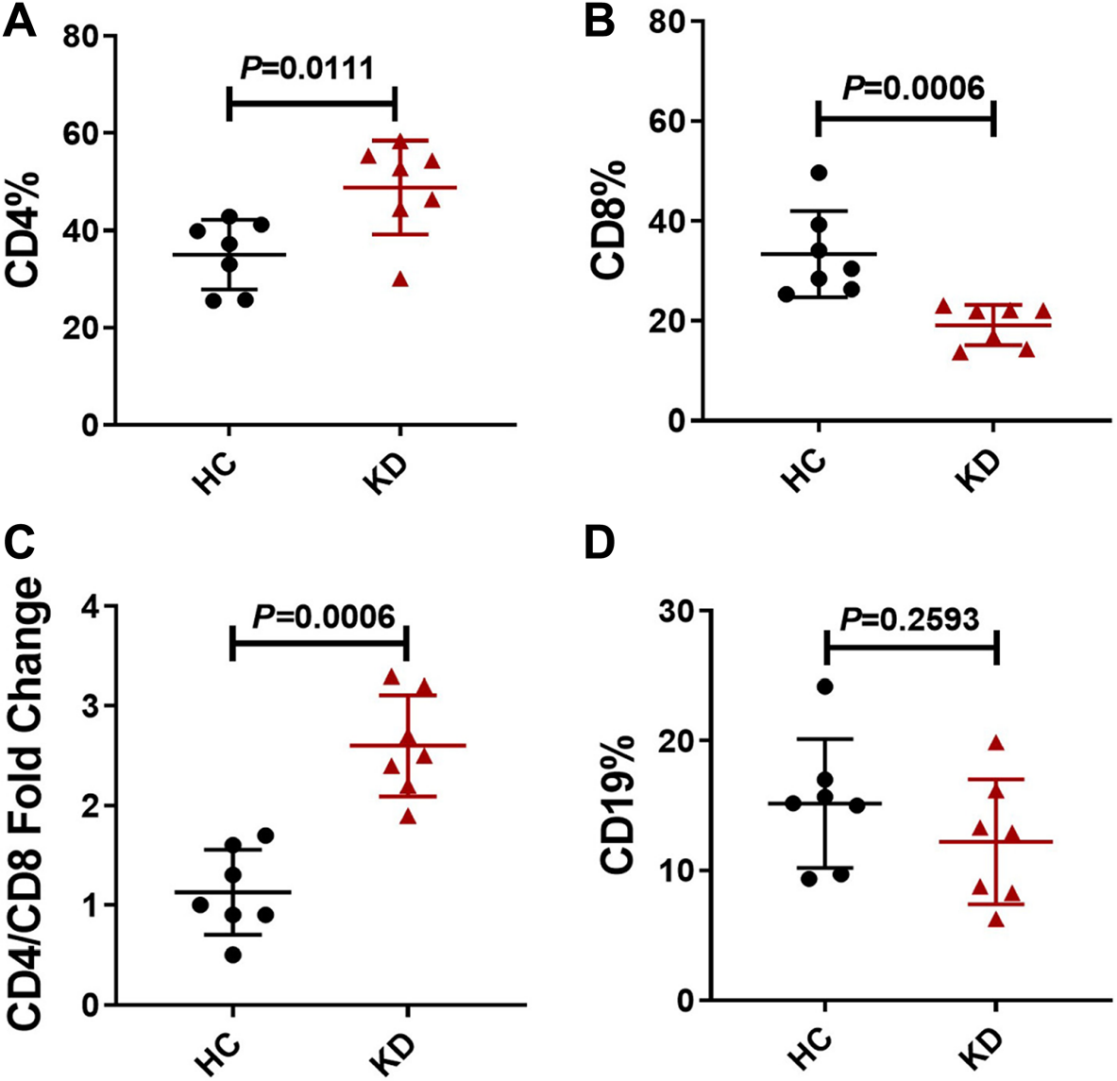
Immune imbalance of peripheral lymphocytes in the acute-phase Kawasaki disease. **a** The percentage of CD4^+^ T lymphocyte in peripheral blood. **b** The percentage of CD8^+^ T lymphocyte in peripheral blood. **c** The ratio of CD4/CD8 in peripheral blood. **d** The percentage of CD19^+^ B lymphocyte in peripheral blood. Peripheral blood was collected and anticoagulated with EDTAs in patients with Kawasaki disease (*n* = 7) and controls (*n* = 7). The percentage of lymphocyte subsets was determined by flow cytometry. Individual level of each subject was shown. The horizon line presented mean, and error bar presented standard deviation. HC, Healthy controls group; KD, Kawasaki disease group. Representative FlowJo plots were shown in supplementary material Fig.S1

We first examined the percentage of lymphocytes in acute-phase Kawasaki disease. Clinical information of the patients was described in Table 1. The flow cytometry results revealed an immune cell imbalance in peripheral lymphocytes in acute-phase Kawasaki disease. In the Kawasaki disease group, the percentage of CD4^+^ T cells was increased and the percentage of CD8^+^ T cells (Fig. 1a and b) decreased relative to the corresponding percentages in controls, leading to a significant increase in the CD4/CD8 ratio in the KD group (Fig. 1c). This result may be associated with disease severity and outcome, although this information has limited clinical value. However, the percentage of CD19^+^ B cells did not significantly differ between the KD and control groups (Fig. 1d). Representative FlowJo plots were shown in supplementary material Fig.S1. These results suggest that an immune imbalance exists in acute-phase Kawasaki disease.

**Table 1:** Clinical parameters of Kawasaki disease patients

Sex	KD1	KD2	KD3	KD4	KD5	KD6	KD7
	Male	Male	Male	Male	Female	Female	Female
Age(y)	3.92	1.25	1.75	2.17	1.17	1.50	1.75
WBC(10^9^/L)	22.67	9.18	12.31	21.78	28.34	20.54	17.76
RBC(10^12^/L)	4.19	4.56	4.24	3.91	3.96	4.03	3.94
Hemoglobin(g/L)	116	116	114	109	105	104	97
PLT (10^9^/L)	535	370	565	602	527	457	454
CRP (mg/L)	67.7	99.8	12.56	174.97	78.7	35.4	37
TBil (umol/L)	5.8	12.5	4.2	10.2	4.7	7.9	4.2
ALT (IU/L)	529	27	12	32	68	14	21
AST (IU/L)	131	31	19	21	35	20	23
Sodium (mmol/L)	134.2	126.7	138.9	130	135.6	138.8	136.9
ESR (mm/h)	63	32	46	58	87	90	89
PCT (ng/ml)	1.75	1.95	0.16	1.26	7.38	0.33	0.16

*KD* Kawasaki disease, *y* Year, *WBC* White blood cell counts, *RBC* Red blood cell counts, *PLT* Platelet, *CRP* C-reactive protein, *TBil* Total bilirubin, *ALT* Alanine aminotransferase, *AST* Aspartate aminotransferase, *ESR* Erythrocyte sedimentation rate, *PCT* Procalcitonin

### Effect of activin a on the expression of ActRIIA on peripheral lymphocytes

**Fig. 2:**
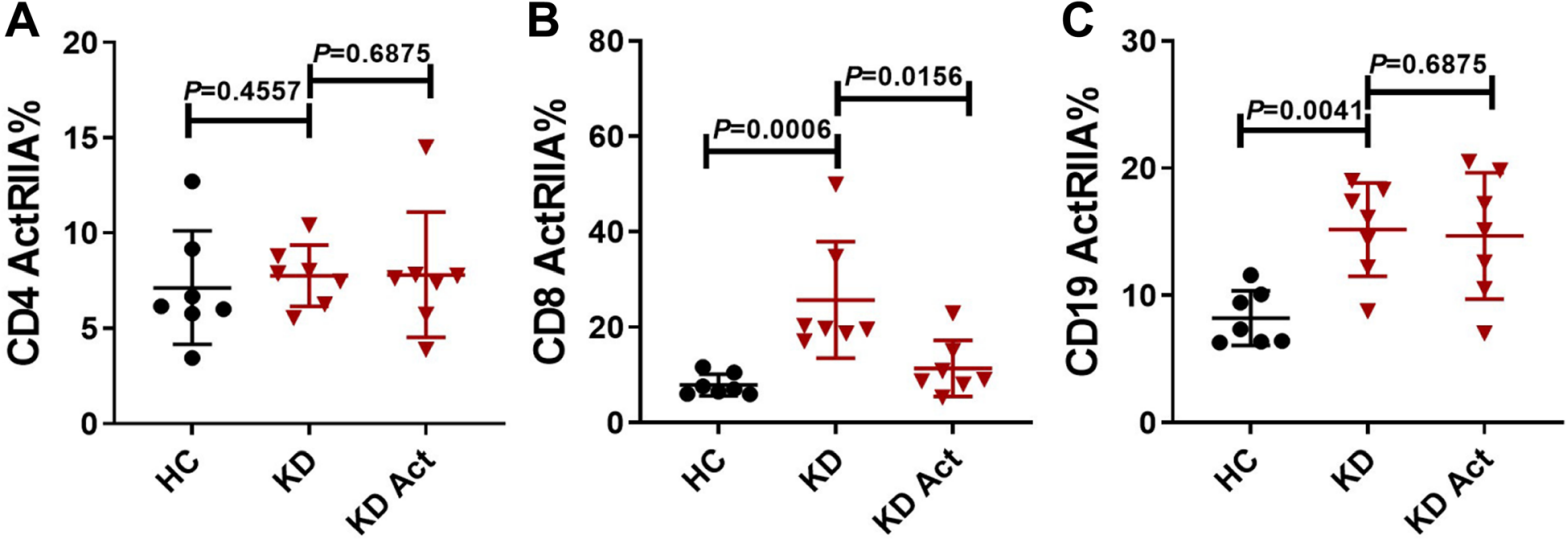
Effect of activin A on the expression of ActRIIA on peripheral lymphocytes. **a** The expression of ActRIIA on CD4^+^ T lymphocyte. **b** The expression of ActRIIA on CD8^+^ T lymphocyte. **c** The expression of ActRIIA on CD19^+^ B lymphocyte. Total PBMCs were isolated from Kawasaki disease patients and healthy controls, stimulated with activin A (5 ng/ml) for 24 h in vitro. Cells were then harvested, stained and the expression of cell surface molecules ActRIIA was analyzed by flow cytometry. Individual level of each subject was shown. The horizon line presented mean, and error bar presented standard deviation. HC, Healthy controls group; KD, Kawasaki disease group: KD Act, Kawasaki disease with activin-A stimulated group. Representative FlowJo plots were shown in supplementary material Fig.S2

ActRIIA is a transmembrane protein expressed on the surface of cell membranes. Activin signaling first activates ActRIIA, which phosphorylates type I receptors and then regulates the transcription of target genes through signal transduction via Smad classical or non-SMAD pathways [11, 12]. The flow cytometry results showed that ActRIIA expression on CD8^+^ T cells and CD19^+^ B cells was increased in the Kawasaki disease group compared with the healthy control group. Interestingly, ActRIIA expression on CD8^+^ T cells was reduced after stimulation with activin A (5 ng/ml, Fig. 2b). ActRIIA did not change its expression significantly on CD4^+^ and CD19^+^ cells after activin A treatment (Fig. 2a and c). Representative FlowJo plots were shown in supplementary material Fig.S2. These results indicate that the expression of ActRIIA differs among subsets of peripheral lymphocytes in acute-phase Kawasaki disease and that activin A has different effects on the expression of ActRIIA in different lymphocytes. The findings indicate that activin A suppresses ActRIIA expression on peripheral CD8^+^ T lymphocyte in acute-phase Kawasaki disease.

### Effect of activin a on the activation of peripheral lymphocytes in acute-phase Kawasaki disease

**Fig. 3:**
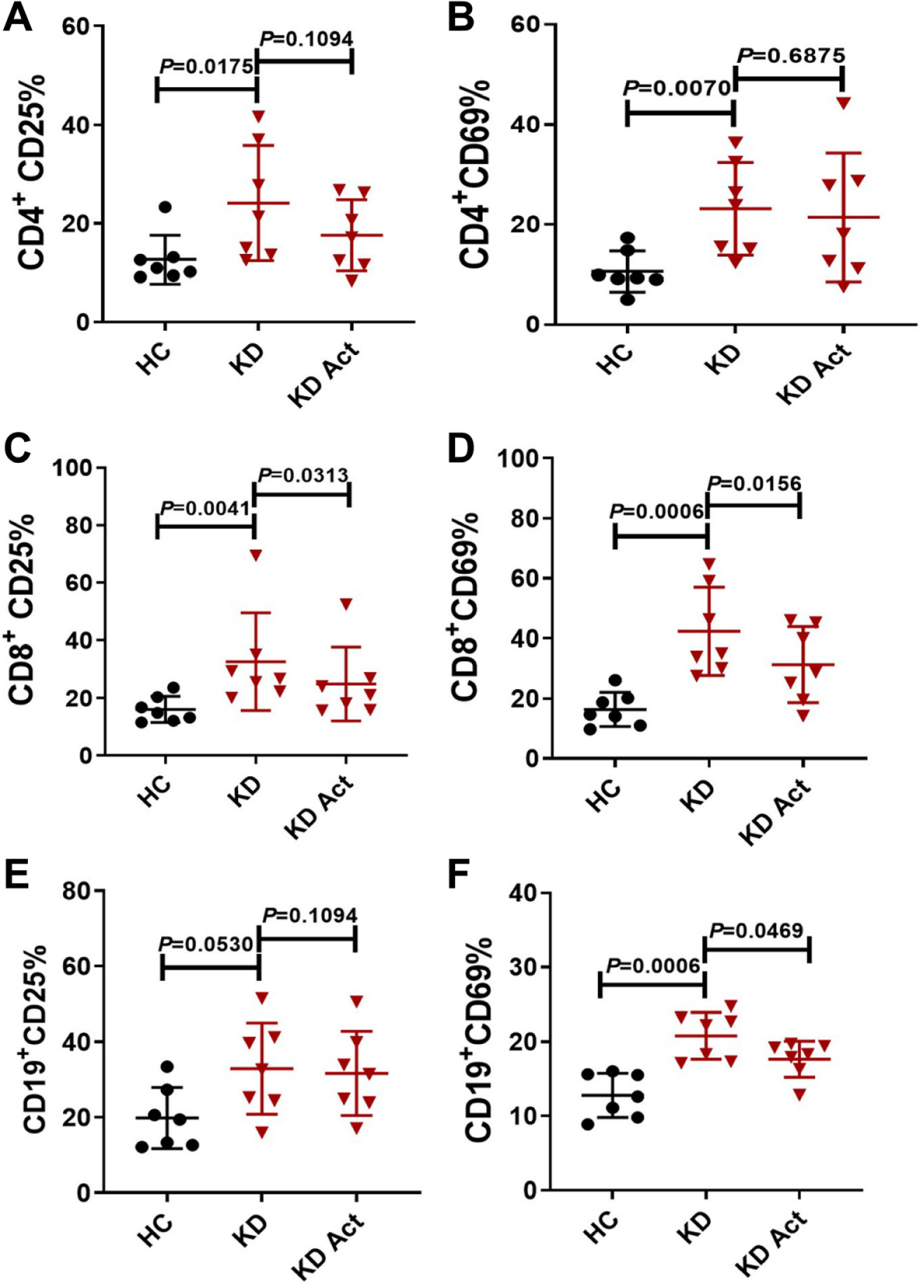
Effect of activin A on activation of peripheral lymphocytes in acute-phase Kawasaki disease. **a** The expression of CD25 on CD4^+^ T lymphocyte. **b** The expression of CD69 on CD4^+^ T lymphocyte. **c** The expression of CD25 on CD8^+^ T lymphocyte. **d** The expression of CD69 on CD8^+^ T lymphocyte. **e** The expression of CD25 on CD19^+^ B lymphocyte. **f** The expression of CD69 on CD19^+^ B lymphocyte. Total PBMCs were isolated from Kawasaki disease patients and healthy controls, stimulated with activin A (5 ng/ml) for 24 h in vitro. Cells were then harvested, stained and the expression of CD25 and CD69 were analyzed by flow cytometry. Individual level of each subject was shown. The horizon line presented mean, and error bar presented standard deviation. HC, Healthy controls group: KD, Kawasaki disease group: KD Act, Kawasaki disease with activin-A stimulated group. Representative FlowJo plots were shown in supplementary material Fig.S3

CD25 is expressed on activated T and B cells. CD69 is involved in the early activation of lymphocytes, monocytes, and platelets. Both of these proteins are considered markers of leukocyte activation [6, 13]. Flow cytometry was used to detect the expression of CD25 and CD69 on the surface of activated peripheral lymphocytes in acute-phase Kawasaki disease. The expression of CD25 and CD69 on lymphocytes was higher in patients with Kawasaki disease than in healthy controls (Fig. 3a-f). Furthermore, the expression of CD25 and CD69 on the surface of CD8^+^ T cells was decreased after stimulation with activin A for 24 h in vitro (Fig. 3c and d). However, no changes in the expression of CD25 and CD69 were evident in CD4^+^ T cells after stimulation with activin A (Fig. 3a and b). In addition, the expression of CD25 on CD19^+^ B cells was not significantly affected by activin A treatment, whereas CD69 expression was downregulated (Fig. 3e and f). Representative FlowJo plots were shown in supplementary material Fig.S3. The above results suggest that peripheral lymphocytes are over-activated in acute-phase Kawasaki disease. Activin A suppressed the activation of CD8^+^ T cells in acute-phase Kawasaki disease.

## Discussion

Peripheral lymphocytes are out of balance in acute-phase Kawasaki disease, and this imbalance is accompanied by abnormal immune activation. Previous studies have shown that activin A activates monocytes/macrophages in the resting state. The activity of monocytes/macrophages that are in an over-active state is often inhibited [10, 14]. However, it has not been reported whether activin A plays a role in the activation of peripheral lymphocytes in acute-phase Kawasaki disease.

Accompanied by peripheral lymphocytes imbalance, immune disorders and abnormal regulation trigger Kawasaki disease. Activated immune cells, such as T cells, B cells, monocytes/macrophages, dendritic cells and mast cells, can synthesize and secrete activin A. Activin A participates in a variety of diseases by exerting autocrine and/or paracrine effects. Previous studies have shown that activin A is closely related to human autoimmune diseases, such as systemic lupus erythematosus and rheumatoid arthritis. The concentration of activin A is increased in the synovial fluid of joints in patients with rheumatoid arthritis [15, 16]. Activin A regulates the proliferation and differentiation of lymphocytes and the re-epithelialization and formation of granulation tissue to participate in wound repair after skin injury [17]. The roles of activin A have been widely studied, and a dual role of activin A has been proposed; its roles may vary depending on the tissue involved and the disease state. These findings suggest that activin A is involved in autoimmune diseases and that its expression correlates with disease activity. However, its effects on KD immunopathology have not been explored. ActRIIA is very important for intracellular signal transduction. Activin A binds with high affinity to ActRIIA.

In this study, the activin signaling pathway was found to be activated in acute-phase Kawasaki disease. ActRIIA was upregulated on CD8^+^ T and CD19^+^ B cells in acute-phase Kawasaki disease. However, the expression of ActRIIA on CD8^+^ T cells was suppressed following stimulation with activin A in vitro. We propose two potential reasons for these observations. One reason is that peripheral lymphocytes are over-activated in acute-phase Kawasaki disease, and activin A may play various roles in different lymphocytes or cell states through autocrine or paracrine modes of action. The second reason is that the sources of activin A in vivo are extensive. Activated peripheral lymphocytes or damaged vascular endothelial cells may also be involved in the secretion of activin A. The exact mechanism is unclear and needs further exploration.

The immune system is dysfunctional in acute-phase Kawasaki disease, in part due to the imbalance in the proportions of immune cells. The proportion of CD4^+^ T cells was increased while that of CD8^+^ T cells was decreased in KD, leading to an imbalance in the CD4/CD8 ratio. However, the proportion of CD19^+^ cells differed little between patients with Kawasaki disease and healthy controls. Thus, lymphocytes regulatory dysfunction and overactivation may cause Kawasaki disease. The activation of peripheral lymphocytes in KD was observed in our study, consistent with previous observations of lymphocyte, neutrophil and monocyte activation in KD [6, 18, 19]. It has been suggested that both innate immunity and adaptive immunity are actively involved in acute-phase Kawasaki disease. ActRIIA is a transmembrane serine-threonine kinase receptor that is primarily involved in ligand binding and includes an extracellular ligand-binding domain, a transmembrane domain and a cytoplasmic serine-threonine kinase domain [20]. The expression of ActRIIA on CD8^+^ T cells and CD19^+^ B cells in Kawasaki disease was increased in the present study. The abnormal activation of peripheral lymphocytes may be related to the abnormal expression of ActRIIA in acute-phase Kawasaki disease. The activity of peripheral lymphocytes was enhanced in the patients with KD, as evidenced by the increased expression of CD25 and CD69 on cells. The expression of CD25 and CD69 was decreased on CD8^+^ T cells after stimulation with activin A in vitro, suggesting that activin A can suppress the activation of peripheral CD8^+^ T lymphocyte.

## Conclusions

In summary, the expression of ActRIIA on peripheral CD8^+^ T lymphocyte was increased in acute-phase Kawasaki disease and was decreased following cell stimulation by activin A in vitro. Peripheral lymphocytes were activated in acute-phase Kawasaki disease, and activin A had different effects on different types of lymphocytes. The findings indicate that activin A suppresses the activity of peripheral CD8^+^ T lymphocyte in acute-phase Kawasaki disease. Whether Kawasaki disease can be alleviated by regulating the expression of ActRIIA on peripheral lymphocytes warrants further investigation. This study provides a new perspective for the treatment of Kawasaki disease.

## Methods

### Patients

Seven patients with Kawasaki disease (4 males and 3 females, 1.93 ± 0.87 years old) in the acute stage who were hospitalized at Shenzhen Children’s Hospital between January 2020 and August 2020 were enrolled in this study, all of whom met the criteria proposed by the Japanese Kawasaki Disease Research Committee [21]. All of the patients were complete Kawasaki disease patients and IVIG responders without coronary artery lesions. The clinical parameters of the Kawasaki disease patients are listed in Table 1. To be diagnosed with complete KD, the patient must have ≥5 days of fever as well as ≥4 of the 5 principal clinical features, which are as follows: (1) mucosal changes: erythaema and cracking of lips, “strawberry tongue” and/or erythaema of the oral and pharyngeal mucosa; (2) conjunctivitis: bilateral bulbar nonexudative conjunctival injection, often limbic sparing; (3) polymorphous rash; (4) extremity changes: erythaema and oedema of the hands and feet; and (5) lymphadenopathy: acute, non-suppurative, cervical lymphadenopathy (1.5 cm diameter), typically unilateral [22, 23]. The acute phase is characterized by high-spiking fevers (typically > 39.0 °C), with the other principal features listed above. Peripheral blood (3 mL) was collected in an EDTA anticoagulation tube in the acute stage of KD (usually 1–11 days into the course of the disease) prior to receiving IVIG treatment. Patients receiving any prior treatment were excluded. Blood was centrifuged, and serum was immediately stored at − 80 °C for later analysis. Seven healthy volunteers (4 males and 3 females, 1.86 ± 0.63 years old) were included as healthy controls. After obtaining informed consent, blood was drawn from the study participants. The study protocol was approved by the Ethics Committee of Shenzhen Children’s Hospital.

### Flow cytometry

Peripheral blood mononuclear cells (PBMCs) were isolated from blood samples anti-coagulated with EDTA using Ficoll-Hypaque density gradient centrifugation. Cells were seeded in 12-well plates at a density of 1 × 10^6^ cells per well. The cells were then grown in complete RPMI 1640 medium supplemented with 2% FCS and stimulated with or without 5 ng/ml activin A for 24 h in a 5% CO2 incubator. To detect the expression of ActRIIA, cells were stained with anti-CD4-eFloure450 (BD Biosciences), anti-CD8-PE-Cy7 (BD Biosciences), anti-CD19-PE (BD Biosciences) and anti-ActRIIA-APC (BD Biosciences) antibodies for 30 min at RT. To detect the expression of CD25 and CD69, cells were stained with anti-CD4-PE-Cy7 (BD Biosciences), anti-CD8-BV510 (BD Biosciences), anti-CD19-BV421 (BD Biosciences), anti-CD25-PE (BD Biosciences) and anti-69-FITC (BD Biosciences) antibodies for 30 min at RT. The labelled cells were analysed by flow cytometry (BD FACS Canto II). The data were collected and analysed with *FlowJo v10* to assess the percentages of fluorescence-positive cells.

### Statistical analysis

The data are expressed as mean ± standard deviation (SD). Frequencies were compared between groups using a Mann-Whitney (nonparametric) test. Statistical evaluation was performed by the statistical software SPSS 17.0. Differences of *P* < 0.05 were considered statistically significant.

## Supplementary Information

**Additional file 1: Fig.S1** The percentage of lymphocyte subsets in the acute-phase Kawasaki disease. Peripheral blood was collected and anticoagulated with EDTAs in patients with Kawasaki disease and healthy controls. The percentage of CD4^+^ T lymphocyte, CD8^+^ T lymphocyte and CD19^+^ B lymphocyte in peripheral blood was determined by flow cytometry. R1 indicates gate for lymphocyte. HC, Healthy controls group; KD, Kawasaki disease group. **Fig.S2** The expression of ActRIIA on CD4^+^ T lymphocyte, CD8^+^ T lymphocyte and CD19^+^ B lymphocyte. Total PBMCs were isolated from Kawasaki disease patients and healthy controls, stimulated with activin A (5 ng/ml) for 24 h in vitro. Cells were then harvested, stained and the expression of cell surface molecules ActRIIA was analyzed by flow cytometry. HC, Healthy controls group; KD, Kawasaki disease group: KD Act, Kawasaki disease with activin-A stimulated group. **Fig.S3** The expression of CD25 and CD69 on CD4^+^ T lymphocyte, CD8^+^ T lymphocyte and CD19^+^ B lymphocyte. Total PBMCs were isolated from Kawasaki disease patients and healthy controls, stimulated with activin A (5 ng/ml) for 24 h in vitro. Cells were then harvested, stained and the expression of CD25 and CD69 were analyzed by flow cytometry. HC, Healthy controls group: KD, Kawasaki disease group: KD Act, Kawasaki disease with activin-A stimulated group.

## Data Availability

The datasets used and analysed during the current study are available from the corresponding author on reasonable request.

## Abbreviations

KD: Kawasaki disease
ActRIIA: Activin type IIA receptor
PBMCs: Peripheral blood mononuclear cells
HC: Healthy control
Act: Activin A
