# Supplementary material for: Activin a suppresses peripheral CD8+ T lymphocyte activity in acute-phase Kawasaki disease
Source: BMC Immunol. 2021 Feb 23;22:17. doi: 10.1186/s12865-021-00407-x (PMC7903692; doi:10.1186/s12865-021-00407-x)
Supplement: Supplementary file 1 — Additional file 1: Fig.S1 The percentage of lymphocyte subsets in the acute-phase Kawasaki disease. Peripheral blood was collected and anticoagulated with EDTAs in patients with Kawasaki disease and healthy controls. The percentage of CD4+ T lymphocyte, CD8+ T lymphocyte and CD19+ B lymphocyte in peripheral blood was determined by flow cytometry. R1 indicates gate for lymphocyte. HC, Healthy controls group; KD, Kawasaki disease group. Fig.S2 The expression of ActRIIA on CD4+ T lymphocyte, CD8+ T lymphocyte and CD19+ B lymphocyte. Total PBMCs were isolated from Kawasaki disease patients and healthy controls, stimulated with activin A (5 ng/ml) for 24 h in vitro. Cells were then harvested, stained and the expression of cell surface molecules ActRIIA was analyzed by flow cytometry. HC, Healthy controls group; KD, Kawasaki disease group: KD Act, Kawasaki disease with activin-A stimulated group. Fig.S3 The expression of CD25 and CD69 on CD4+ T lymphocyte, CD8+ T lymphocyte and CD19+ B lymphocyte. Total PBMCs were isolated from Kawasaki disease patients and healthy controls, stimulated with activin A (5 ng/ml) for 24 h in vitro. Cells were then harvested, stained and the expression of CD25 and CD69 were analyzed by flow cytometry. HC, Healthy controls group: KD, Kawasaki disease group: KD Act, Kawasaki disease with activin-A stimulated group. [file 12865_2021_407_MOESM1_ESM.doc]

**Activin A suppresses peripheral CD8^+^ T lymphocyte activity in acute-phase Kawasaki disease**

Qian Wu^1,2^, Ruohang Weng^2^, Yongbin Xu^2^ Linlin Wang^2^, Yanyan Huang^2^, Jun Yang^2*^

*Correspondence to rogasansz@163.com

Supplementary materials


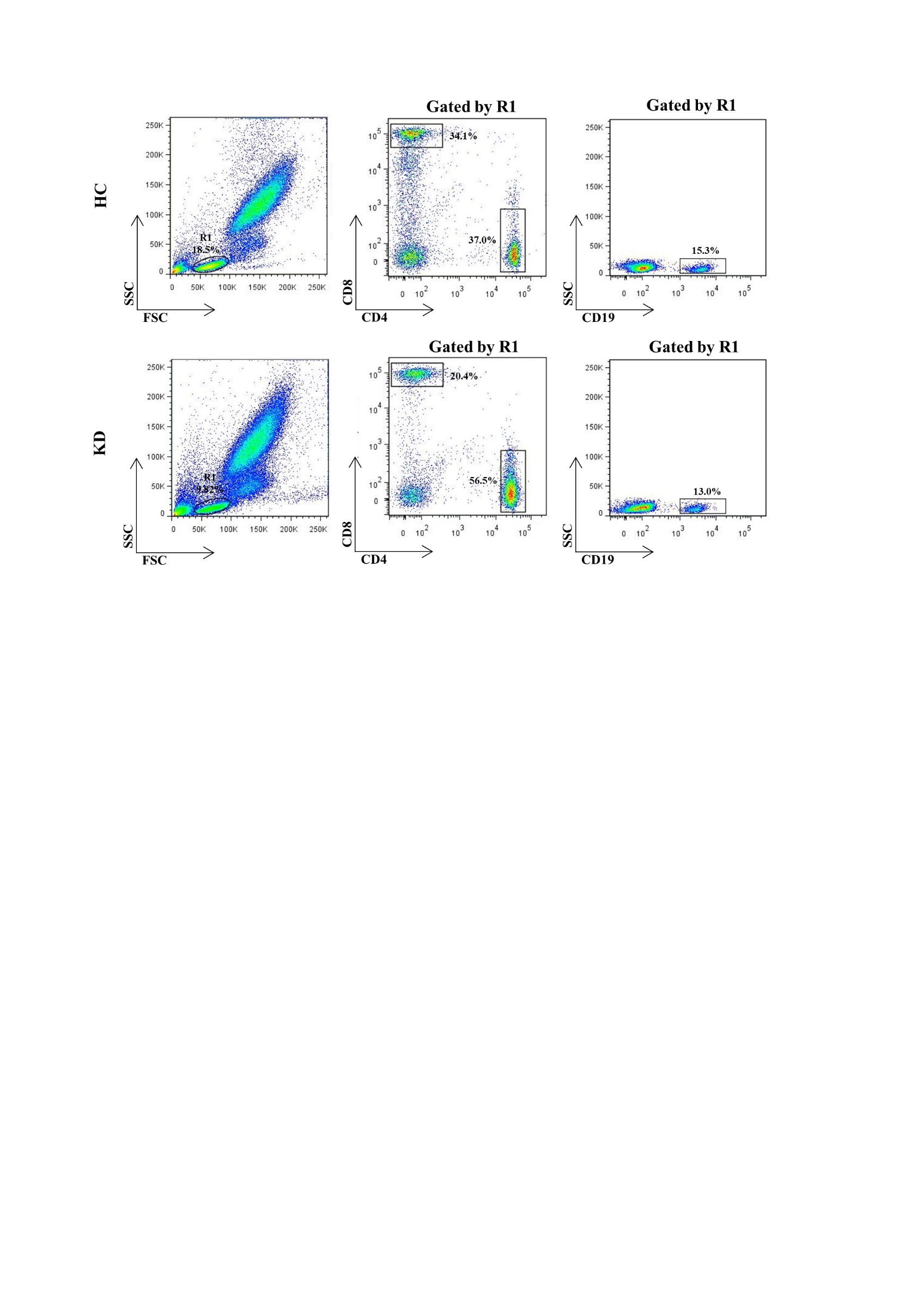


**Fig.S1** The percentage of lymphocyte subsets in the acute-phase Kawasaki disease. Peripheral blood was collected and anticoagulated with EDTAs in patients with Kawasaki disease and healthy controls. The percentage of CD4^+^ T lymphocyte, CD8^+^ T lymphocyte and CD19^+^ B lymphocyte in peripheral blood was determined by flow cytometry. R1 indicates gate for lymphocyte. HC, Healthy controls group; KD, Kawasaki disease group.


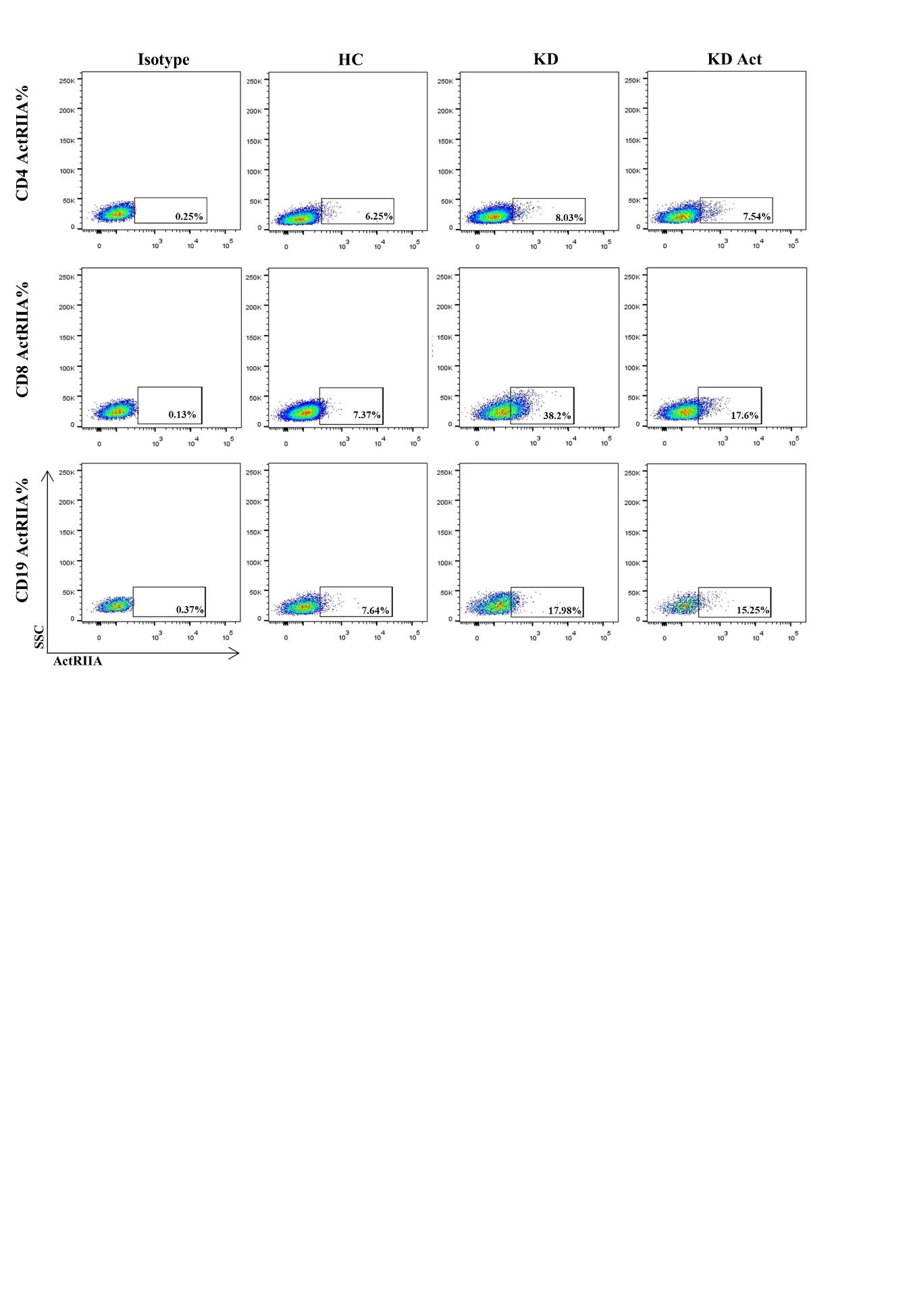


**Fig.S2** The expression of ActRIIA on CD4^+^ T lymphocyte, CD8^+^ T lymphocyte and CD19^+^ B lymphocyte. Total PBMCs were isolated from Kawasaki disease patients and healthy controls, stimulated with activin A(5ng/ml) for 24h *in vitro*. Cells were then harvested, stained and the expression of cell surface molecules ActRIIA was analyzed by flow cytometry. HC, Healthy controls group; KD, Kawasaki disease group: KD Act, Kawasaki disease with activin-A stimulated group.


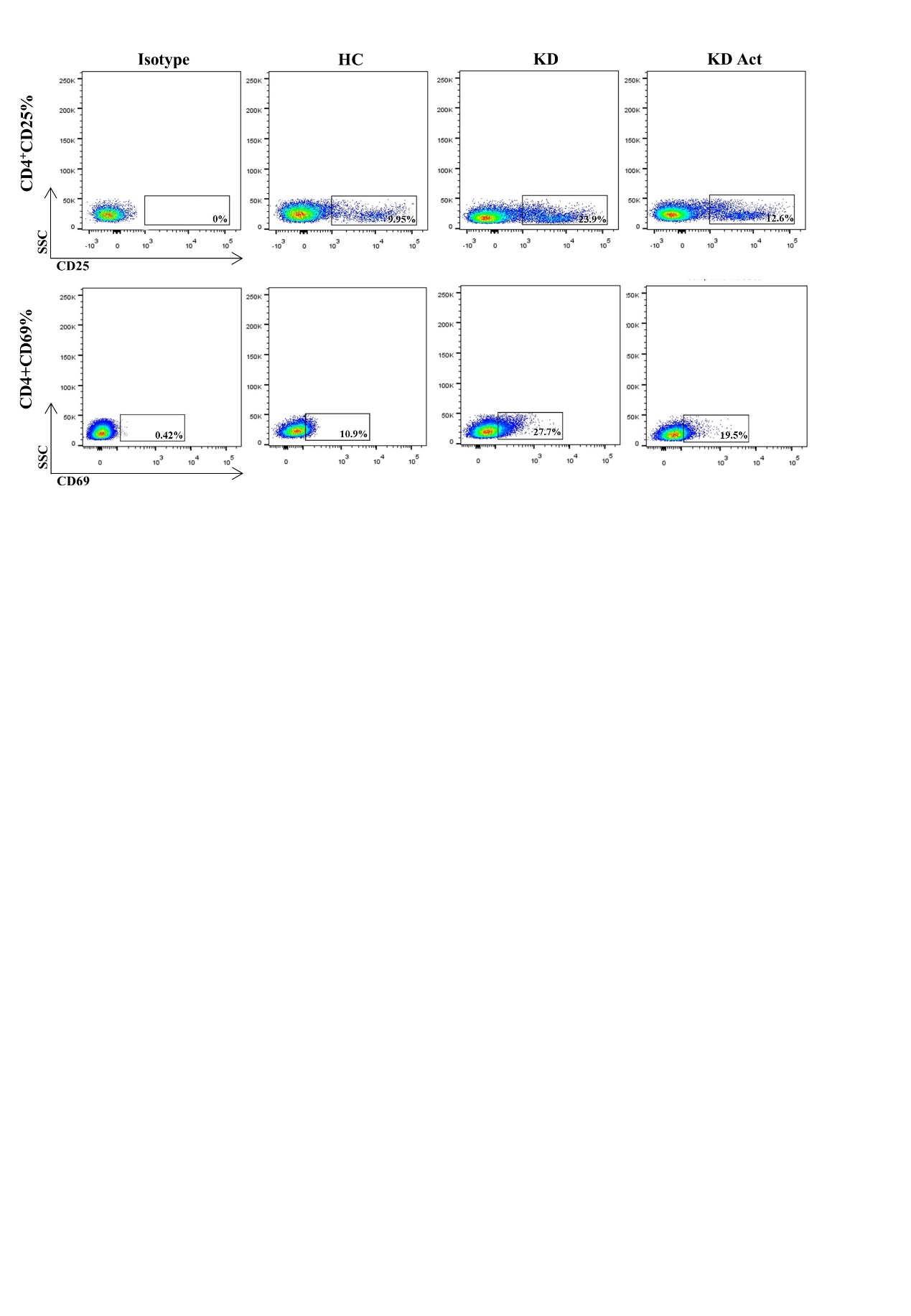

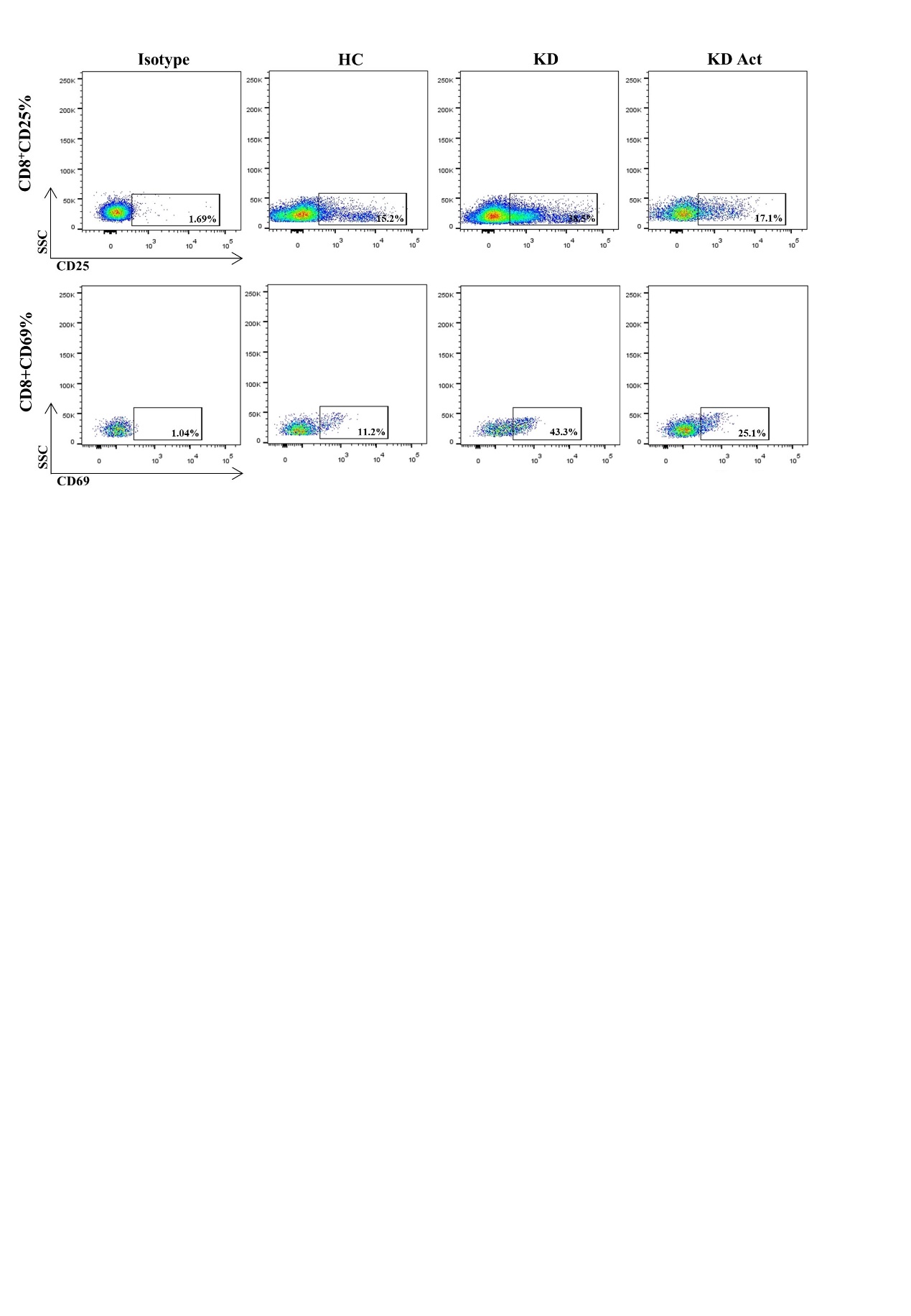


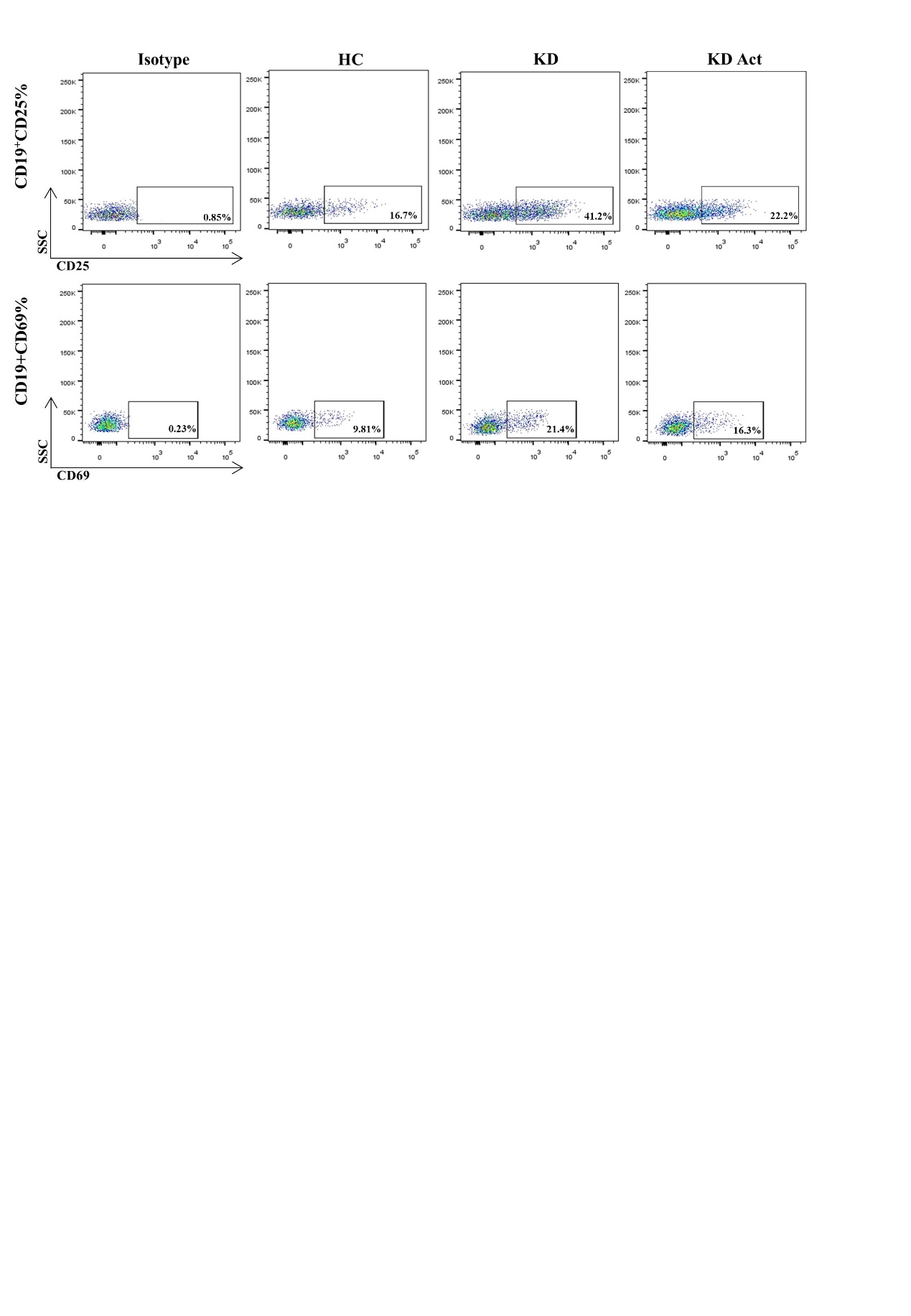


**Fig.S3** The expression of CD25 and CD69 on CD4^+^ T lymphocyte, CD8^+^ T lymphocyte and CD19^+^ B lymphocyte. Total PBMCs were isolated from Kawasaki disease patients and healthy controls, stimulated with activin A(5ng/ml) for 24h *in vitro*. Cells were then harvested, stained and the expression of CD25 and CD69 were analyzed by flow cytometry. HC, Healthy controls group: KD, Kawasaki disease group: KD Act, Kawasaki disease with activin-A stimulated group.
